# The Symptomatology of Cerebellar Tumours

**Published:** 1905-05-13

**Authors:** 


### THE SYMPTOMATOLOGY OF CEREBELLAR TUMOURS.

In a valuable communication Drs. Grainger Stewart and Gordon Holmes[1] have set out the result of an investigation of 40 cases of cerebellar tumour observed at the Hospital for the Paralysed and Epileptic in Queen Square. The cases are divided into two classes—namely, (1) intra-cerebellar, and (2) extra-cerebellar, situate, that is to say in the angle between the cerebellum and the medulla oblongata.

### General Symptoms.

Headache is a constant feature. It is severe in character, and chiefly occipital. Vomiting is almost invariable. Optic neuritis is constant, early and intense.

### Special Symptoms.

Vertigo is almost constant, and requires to be carefully analysed, for it supplies evidence of localisation. Thus, in both extra- and intra-cerebellar growths the sense of displacement of external objects is from the side of the lesion to the healthy side. But as regards the subjective rotation of self, the sense of displacement varies with the situation of the mass. Thus in intra-cerebellar cases the subjective rotation of self is from the side of the lesion to the healthy side—that is to say, in the same direction as the apparent displacement of external objects, while in the case of extra-cerebellar growths the apparent displacement of self is from the healthy side to the side of the lesion or in a direction opposite to that of the apparent movement of external objects.

Deafness is common in extra-cerebellar cases, rare in the other variety. Tinnitus is fairly common in the former and is then always referred to the same side as the lesion. In the latter, if present, it is slight and generalised.

### Cranial Nerve Symptoms.

A majority of cases of both kinds develop some paresis of the external rectus on the side of the lesion. Nystagmus is a most useful localising symptom for both varieties. It consists of a slow, deliberate, jerking movement towards the side of the lesion when the eyes are turned in that direction and is most marked on lateral movement.

The facial and trigeminal nerves are occasionally affected in both classes of case, but more often in the extra-cerebellar. The glosso-pharyngeal, vagus, and hypoglossal are rarely affected.

### Motor Symptoms.

In the intra-cerebellar form paresis of the same side as the lesion is well marked, while in the other variety such weakness is far less, if present at all; but in this form spasticity of the opposite side is frequent.

Ataxia is a classical symptom. It affects chiefly the side of the lesion, and consists of a disturbed association of movements; it appears only on active movement, and is not increased by closing the eyes; that is to say, it is central in origin, and not, like the ataxia of tabes, peripheral. The symptom described by Babinski under the term "diadococinesia," and purporting to be pathognomonic of cerebellar tumour, consists in an inability to perform rapid alternate movements, such as rapid pronation and supination of the hand. The authors do not consider it pathognomonic. A common but not constant attitude consisted of a flexion of the head towards the side of the lesion, with the point of the chin pointing to the opposite shoulder.

The staggering gait associated with the disease consists of two elements: (1) A tendency to stumble toward the side of the lesion; (2) a tendency to deviate from the desired direction towards the affected side. In a chronic case this leads to a voluntary correction in the opposite direction, which may be over-done, and, if its true significance is unappreciated, may lead to localisation of the tumour on the wrong side.

The deep reflexes are very variable. The extensor plantar reflex is never found in uncomplicated cases of intra-cerebellar tumour, but may appear in the extra-cerebellar form when there is pressure on the pons.

[1] Brain. Winter Quarter, 1904.

